# Relation of Ozone to Disease

**Published:** 1874-11

**Authors:** 


THE RELATION OF OZONE TO DISEASE—*By Dr. Baldwin.*—* * * When I commenced the study of this subject, I was biased in favor of the view that ozone could produce disease directly by its presence, and indirectly by its absence. But after a careful and candid investigation, I think this view entirely erroneous. Reasoning *a priori*, from the premises furnished by what I found known of ozone and of epidemics, did not result in a conclusion favorable to any such hypothesis; while a resort to recorded observations proved no more satisfactory. It is true that occasionally, in some circumscribed locality, the fluctuations of an epidemic have seemed to sustain a certain relationship to the fluctuations in the amount of ozone; but such an exception proves nothing. In truth, it would be strange if such a coincidence did not sometimes occur; for, by a well-known law, a parallelism must exist, now and then, between two independent and irregular curves.

In the relation of ozone to disease, that which accords perfectly with the known properties of ozone, which harmonizes with the results of all observations, and which at once challenges rational belief, seems to be simply this: *ozone influences the general health only in so far as it purifies the air by destroying*—not the the living germs of disease—*but the products of decomposition.* Beyond this, all views concerning the action of ozone, as a cause, a remedy, or a preventive of disease, rest upon vague and unfounded hypothesis.

